# Severe Morbidity According to Sex in the Era of Combined Antiretroviral Therapy: The ANRS CO3 Aquitaine Cohort

**DOI:** 10.1371/journal.pone.0102671

**Published:** 2014-07-30

**Authors:** Mojgan Hessamfar, Céline Colin, Mathias Bruyand, Madeleine Decoin, Fabrice Bonnet, Patrick Mercié, Didier Neau, Charles Cazanave, Jean-Luc Pellegrin, François Dabis, Philippe Morlat, Geneviève Chêne, the GECSA study group

**Affiliations:** 1 INSERM U897 & CIC-EC7, Université de Bordeaux, Institut de Santé Publique Epidémiologie et Développement (ISPED), Bordeaux, France; 2 Centre Hospitalier Universitaire (CHU) Bordeaux, Coordination Régionale de la lutte contre l'infection à VIH (COREVIH) Aquitaine, Bordeaux, France; 3 CHU Bordeaux, Service de Médecine Interne et Maladies Infectieuses, Bordeaux, France; 4 CHU Bordeaux, Service de Médecine Interne et Immunologie Clinique, Bordeaux, France; 5 CHU de Bordeaux, Fédération des Maladies Infectieuses et Tropicales, Bordeaux, France; 6 Université de Bordeaux, Bordeaux, France; Public Health Agency of Barcelona, Spain

## Abstract

**Objective:**

To describe trends and determinants of severe morbidity in HIV-infected women and men.

**Design:**

A French prospective cohort of HIV-infected patients of both sexes and all transmission categories.

**Methods:**

We used hospital admission data from January 2000 to December 2008. A severe morbid event (SME) was defined as a clinical event requiring hospitalization for ≥48 h, several events could be reported during hospitalization. Yearly incidence rates of SME were estimated and compared using Generalized Estimating Equations.

**Results:**

Among 4,987 patients (27% women), followed for a median of 8.7 years, 1,473 (30%) were hospitalized (3,049 hospitalizations for 5,963 SME). The yearly incidence rate of hospitalization decreased in men, from 155 in 2000 to 80/1,000 person-years (PY) in 2008 and in women, from 125 to 71/1,000 PY, (p<0.001). This trend was observed for all SME except for hepatic events, stable in men (15 to 13/1,000 PY) and increasing in women (2.5 to 11.5), cardiovascular events increasing in men (6 to 10/1,000 PY) and in women (6 to 14) and non-AIDS non-hepatic malignancies increasing in men (4 to 7/1,000 PY) and stable in women (2.5). Intraveneous drug users, age >50 years, HIV RNA >10,000 copies, CD4 <500/mm^3^, AIDS stage, hepatitis C co-infection and cardiovascular risk factors (diabetes, high blood pressure, and tobacco use) were associated with SME.

**Conclusions:**

HIV-infected individuals in care in France require less and less frequently hospitalization. Women are now presenting with severe hepatic and cardio-vascular events. Disparities in SME between men and women are primarily explained by different exposure patterns to risk factors. Women should be targeted to benefit cardiovascular prevention policies as well as men.

## Introduction

Nearly 55% of adults living with HIV across the world are women, this proportion varying from 27% in Western Europe and North America to 58% in Sub-Saharan Africa [1]. This worldwide trend to the feminisation of the epidemic is explained by a more frequent transmission of HIV through heterosexual intercourse.

Since the advent of combination antiretroviral therapy (cART), HIV-infected patients are living longer [2] and free from AIDS-related diseases [3]. However, as their life expectancy increases, HIV-infected patients are exposed to new complications related to ageing, long term exposure to treatment, and chronic co-morbidities [4], [5].

The introduction of cART considerably decreased the overall severe morbidity, as reflected by less frequent inpatient health care utilization [6]. In recent years, however, the decrease in hospitalization rates has been less dramatic and at times stagnant [6]–[8]. This evolution may be explained by the ageing of the HIV-infected population [9], an increase in complications due to comorbid diseases, and side effects of cART [10]–[13]. Furthermore, the above mentioned evolution has not been uniform, with women, and older patients experiencing severe complications more frequently than others [14], [15].

We have investigated the causes of death of HIV-infected women in comparison with men, through a national survey in France [16]. Women died more often from AIDS and men from causes unrelated to AIDS. So far, knowledge is limited on potential sex disparities in severe morbidity of these patients and their consequences for the case management, especially in a context of universal access to care.

The aim of the current study was to describe trends and determinants of severe morbidity according to sex in HIV-infected patients, by examining hospital admissions among a prospective cohort of HIV-infected patients over a 9-year period of the cART era.

## Methods

### Study design

This is an observational study within the Aquitaine CO3 open Cohort of all HIV-infected patients addressed to a public health care center in the Aquitaine region (South-Western France) and followed prospectively.

### Patients

#### Ethics statement

All patients included in this study gave written informed consent. The study protocol was approved by the Ethics committee of Bordeaux University Hospital (Comité de protection des personnes).

#### Aquitaine Cohort

The ANRS CO3 Aquitaine Cohort is an open cohort, initiated in 1987, at the Bordeaux University hospital and eight other public hospitals in the Aquitaine region (South-Western France) by the Groupe d'Epidémiologie Clinique du Sida en Aquitaine (GECSA). Details of the cohort have been reported elsewhere [17]. All adult in- or out-patients of the participating hospital wards who had HIV-1 infection confirmed by Western Blot testing and who had provided informed consent, with at least one follow-up visit after enrolment or a documented date of death, were eligible in the cohort.

### Data collection

A standardized questionnaire collecting data on epidemiological factors, clinical events laboratory measurements and therapeutic interventions is completed by physicians and research nurses at each contact. All events are coded according to the International Classification of Diseases 10^th^ revision [ICD10]. All events retrieved from the database for this analysis have been systematically reviewed and validated by a specialist in the field of HIV infection.

### Definitions of variables

A severe morbid event was defined as a clinical diagnosis associated with a hospitalization stay ≥48 hours, occurring between January 1^st^ 2000 and December 31^st^ 2008. Minor clinical symptoms associated with hospitalization and not leading to a clinical diagnosis at hospital discharge (i.e. nausea, headache, abdominal pain, general symptoms, and more generally R codes of the ICD 10), were not considered as severe morbidity in the analysis. Moreover, scheduled hospitalizations for check-up or diagnostic investigation, systematic control or treatment renewal (Z codes in the ICD10) were also not considered as severe morbidity.

As one hospitalization could be associated with several clinical diagnoses, each different diagnosis was taken into account.

Morbidities were classified as follows: AIDS events [18], bacterial, viral and parasitical infections, psychiatric, cardiovascular, hepatic, gastro-intestinal, respiratory, neurological, haematological, kidney, urological, and dermatological events, non-AIDS-non-hepatic malignancies, and others.

Age was divided into three categories: 18–39, 40–49 and ≥50 years old. Region of origin was classified in three categories: Europe, sub-Saharan Africa, other. HIV transmission group was categorized in four categories: men who have sex with men (MSM), injection drug users (IDU), heterosexuals, others or undetermined. Hepatitis B virus (HBV) infection was defined by at least one positive hepatitis B surface (HBs) antigen measurement and hepatitis C virus (HCV) infection by at least one positive HCV-antibody since inclusion in the cohort.

Diabetes mellitus was defined by at least two glycemia >7 mmol/L during follow-up or by any use of antidiabetic drug. Hypercholesterolemia was defined by high plasma cholesterol >6.24 mmol/L on at least two consecutive measures or any use of lipid-lowering drugs. Hypertriglyceridemia were defined by a plasma level >2.2 mmol/L at two consecutive measures or any use of lipid-lowering drugs. Dyslipidemia is defined as the presence of rather hypercholesterolemia or hypertriglyceridemia. Arterial high blood pressure was defined by at least two consecutive measurements of blood pressure ≥14/9 cm Hg or any use of antihypertensive drugs.

Tobacco use was defined as smoking indication at any time of the follow-up.

Plasma HIV-1 RNA was categorized as <500, 500–9,999, 10,000–99,999 and ≥100,000 copies/ml. CD4 count was categorized as <50, 50–199, 200–499 and ≥500 cells/mm^3^.

For patients diagnosed with severe morbidity, the last CD4 count and HIV1-RNA measurement before the event were considered in the analysis.

cART was defined as the concomitant use of three antiretroviral drugs and categorized in three groups: two nucleoside reverse transcriptase inhibitors (NRTI) + one protease inhibitor (PI), two nucleoside reverse transcriptase inhibitors (NRTI) + one non-nucleoside reverse transcriptase inhibitors (NNRTI), or three NRTIs.

AIDS stage was defined according to the US Centres for Disease Control and Prevention (CDC) classification [18].

### Data analysis

We compared men to women for first values obtained in each calendar year. Demographic, clinical and immuno-virological characteristics including age, region of origin, HIV transmission group, AIDS stage, CD4 count, HIV-1 RNA level, use and type of cART, hepatitis B and C status, presence of dyslipidemia (hypercholesterolemia, hyper triglyceridemia), diabetes mellitus, high blood pressure and tobacco use were described.

We compared patients' characteristics between men and women at hospitalization, by statistical tests for independent samples using nonparametric methods when appropriate.

Descriptive analyses of detailed severe morbidities in each category, according to sex were performed. Yearly rates of patients hospitalized and hospitalizations were calculated by dividing the number of patients hospitalized and hospitalizations by the total number of patients actively followed in each year. Each patient contributed to multiple years of observations, one for each calendar year. Patients could enroll in the cohort at any time preceding or during the study period (January 1^st^, 2000 to December 31^st^, 2008), and thus the number of person-years (PY) was not constant across patients or years. Within each year, we calculated the number of days of follow up. If a patient was enrolled in a given year, the number of days prior to enrolment was excluded from the follow up on that year. If a patient died in a given year, the follow-up was censored on the date of death.

Yearly incidence rates were calculated according to the number of patients actively followed in each specific year and for each diagnosis, separately for men and women. Poisson regression test for trends across calendar years was performed.

Univariate and multivariate analyses of the associations between potential determinants and the presence of a severe morbidity between 2000 and 2008 were performed using a marginal logistic regression with a generalized estimation equations (GEE) approach. The GEE model considers longitudinal updated variables and takes into account repeated measures in each patient. GEE models use an autoregressive correlation matrix and robust standard errors to adjust for the clustered nature of the data here represented by multiple observation years for each patient (assumption of independence between clusters). The specified working correlation structure (i.e. the auto-regressive correlation matrix) describes how the responses (annual rate of events) within clusters (patients) are related to each other. GEE models have been extensively used in data in which the responses are correlated [19], [20].

Variables included in the model were calendar year, sex, transmission group, age, HIV RNA, CD4 cell count, cART use, AIDS stage, dyslipidemia, diabetes, high blood pressure, hepatitis B and C, and tobacco use.

Sensitivity analyses taking into account interactions between sex and different determinants of severe morbidity were also performed.

All analyses were performed with SAS software, version 9,2 (SAS Institute, Cary, North Carolina, USA).

## Results

Between 2000 and 2008, 4,987 HIV-infected patients had at least one contact reported in the ANRS CO3 Aquitaine Cohort, including 1,346 women (27%). The number of patients reporting at least one contact per year steadily increased from 3,079 in 2000 to 3,820 in 2008. The proportion of women remained unchanged from 2000 to 2008 (27%).

The median time of follow-up since the baseline of the cohort was 8.7 years and 6.9 years since the beginning of the study period (2000), representing 21,718 PY of follow-up for men and 8,162 for women.

During the study period, 418 (8.4%) patients were lost to follow-up (no difference between men and women).

### Characteristics of the population

Characteristics and risk factors of co-morbidities of all patients for whom at least one contact had been reported in the ANRS CO3 Aquitaine Cohort between 2000 and 2008 are presented in Table 1.

**Table 1:** Demographic, clinical and immuno-virological characteristics according to sex, by calendar year 2000, 2004 and 2008.

Characteristics	2000 N = 3114		2004 N = 3611		2008 N = 4173		Overall N = 4987	
	Men	Women	Men	Women	Men	Women	Men	Women
**N (%)**	**2279**	**835**	**2622**	**989**	**3006**	**1167**	**3641**	**1346**
**Age (yrs)**								
Median	39	37	43	40	46	40	43	33
18–39	1260 (56)	558 (68)	956 (37)	491 (51)	613 (22)	361 (35)	1891 (52)	883 (65)
40–49	673 (30)	176 (21)	1073 (41)	336 (35)	1297 (47)	477 (45)	1146 (31)	306 (23)
≥50	322 (14)	90 (11)	565 (22)	137 (14)	859 (31)	213 (20)	604 (17)	157 (12)
**Region of Origin**								
Europe	2166 (96)	740 (90)	2483 (96)	791 (82)	2638 (95)	816 (78)	3465 (95)	1058 (79)
S-S Africa	69 (3)	72 (9)	87 (3)	154 (16)	107 (4)	215 (20)	140 (4)	263(19)
Others	20 (1)	12 (1)	24 (1)	19 (2)	24 (1)	20 (2)	36 (1)	25 (2)
**Transmission group**								
MSM	1122 (50)	-	1368 (53)	-	1529 (55)	-	1529 (55)	-
Heterosexual	355 (16)	347 (42)	458 (18)	495 (51)	545 (20)	612 (58)	545 (20)	612 (58)
IDU	598 (26)	382 (46)	565 (22)	368 (38)	481 (17)	339 (32)	481 (17)	339 (32)
Others	180 (8)	95 (12)	203 (8)	101 (11)	214 (8)	100 (10)	214 (8)	100 (10)
**AIDS Stage**	537 (24)	137 (17)	624 (24)	169 (17)	658 (24)	192 (18)	1010 (28)	282 (21)
**CD4 (/mm^3^)**								
Median	414	449	438	449	504	502	445	456
≥500	722 (32)	300 (36)	924(36)	358 (37)	1332 (48)	489 (47)	1460 (40)	561 (42)
200–499	918 (41)	337 (41)	1081 (42)	412 (43)	1059 (38)	402 (38)	1688 (47)	646 (49)
50–199	269 (12)	71 (9)	238 (9)	76 (8)	191 (7)	67 (6)	364 (10)	107 (8)
<50	72 (3)	22 (3)	72 (3)	18 (2)	31 (1)	17 (2)	101 (3)	18 (1)
Missing	274 (12)	94 (11)	279 (11)	100 (10)	150 (5)	76 (7)	-	-
**HIV RNA (cp/ml)**								
Median	1000	630	100	250	40	40	60	80
<500	895 (40)	349 (42)	1362 (53)	481 (50)	2047 (74)	769 (73)	2258 (63)	855 (64)
500–9999	574 (26)	219 (27)	399 (15)	218 (23)	250 (9)	99 (9)	766 (21)	339 (25)
10000–99999	340 (15)	110 (13)	380 (15)	117 (12)	219 (8)	73 (7)	463 (13)	114 (9)
≥100000	170 (7)	53 (6)	168 (6)	47 (5)	99 (4)	33 (3)	121 (3)	27 (2)
Missing	276 (12)	93 (11)	285 (11)	101 (10)	154 (6)	77 (7)	-	-
**cART**								
PI-containing cART	980 (43)	261 (32)	939 (36)	516 (49)	1435 (52)	287 (30)		
Other cART	695 (31)	257 (31)	1002 (39)	320 (30)	859 (31)	379 (39)		
No cART	580 (26)	306 (37)	653 (25)	215 (21)	475 (17)	298 (31)		
**Hepatitis C antibody**	634 (28)	305 (37)	679 (26)	326 (34)	615 (22)	303 (29)	886 (24)	416 (31)
**Hepatitis B (positive HBs antigen)**	171 (8)	22 (3)	195 (8)	36 (4)	216 (8)	45 (4)	297 (8)	57 (4)
**Dyslipidemia^1^**	512 (23)	134 (16)	1050 (41)	298 (31)	1722 (62)	516 (49)	2162 (59)	633 (47)
**Hyper-cholesterolemia^2^**	327 (15)	88 (11)	633 (24)	180 (19)	1060 (38)	356 (34)	1457 (40)	447 (33)
**Hyper-triglyceridemia^3^**	424 (19)	104 (13)	881 (34)	229 (24)	1545 (56)	395 (38)	1894 (52)	483 (36)
**Diabetes Mellitus^4^**	85 (4)	16 (2)	207 (8)	36 (4)	343 (12)	56 (5)	429 (12)	79 (6)
**High blood pressure^5^**	22 (1)	2 (0)	155 (6)	35 (4)	445 (16)	137 (13)	512 (14)	142 (11)
**Tobacco use (any time)**	1340 (59)	454 (55)	1623 (63)	537 (56)	1687 (61)	561 (53)	2093 (58)	662 (49)

1.Hyper-cholesterolemia or hyper-triglyceridemia, 2. Plasma cholesterol >6.24 mmol/L or any use of lipid-lowering drugs, 3. Plasma triglycerides >2.2 mmol/L or any use of lipid-lowering drugs, 4. glycemia >7 mmol/L or any use of antidiabetic drugs, 5. Blood pressure ≥14/9 cm Hg or any use of antihypertensive drugs

For each year, median age was lower for women and increased from 2000 (37 years) to 2008 (43 years). In 2008, patients aged more than 50 years represented more than 30% both in women and men. Patients from sub-Saharan Africa were more often women and their proportion increased through years from 9 to 20%. Women, not concerned by the homosexual group of transmission, were proportionally more often intravenous drug user (IDU) than men. In both sex groups the proportion of IDU decreased overtime, passing for women from 46 to 32% and for men from 26 to 17%. The proportion of heterosexual group of transmission increased over time for both sex groups. Women were less likely to be at the AIDS stage than men, with no trend along the study period (18%). Overall, no differences in CD4 cell count and HIV RNA emerged between sexes. Median CD4 count increased and HIV RNA decreased through years. On average, women were less exposed to cART throughout the study period even if the proportion of women without cART decreased from 37 to 21% between 2000 and 2008.

Women were more frequently infected by hepatitis C virus than men and their proportion decreased over time (37 to 29%). For all cardiovascular risk factors they were less frequently exposed than men. However the proportion of women with dyslipidemia increased from 16 to 49% over the study period and those with high blood pressure from 0 to 13%. Tobacco use was steady through years, more than a half of patients smoked, women less than men.

### Severe morbidity

Overall, 1,473 patients (30%) were hospitalized at least once (27% of the women and 30% of the men of the cohort), resulting in 3,049 hospitalizations for severe morbidity. These hospitalizations accounted for 5,963 severe morbid events, 4,361 in men and 1,602 in women.

The yearly rate of hospitalized patients decreased, from 10% in 2000 to 5% in 2008. This halving occurred both in women (from 8% to 4%) and in men (11% to 5%).

**Figure 1:**
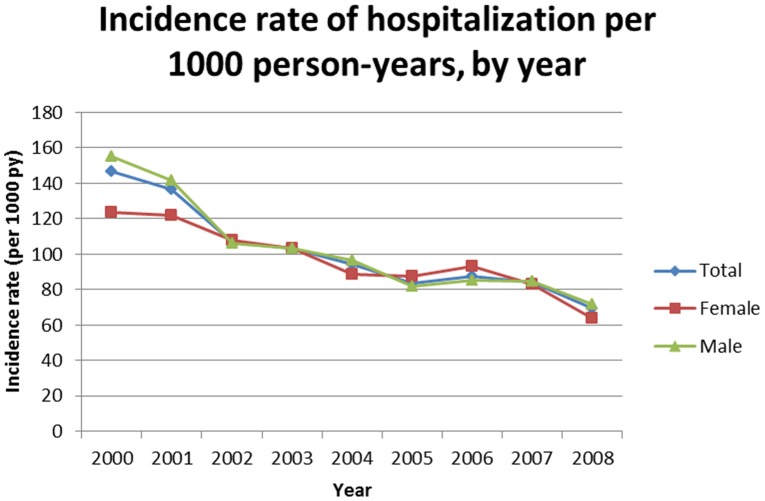
Evolution of yearly incidence rates of hospitalization according to sex (ANRS CO3 Aquitaine Cohort 2000–2008).

Overall, yearly incidence rates of hospitalization decreased, from 147 per 1000 PY in 2000 to 77 per 1000 PY in 2008. The difference between men and women in 2000 (155 vs. 125 per 1000 PY), tended to vanish from 2002 onwards (Fig. 1).

**Figure 2:**
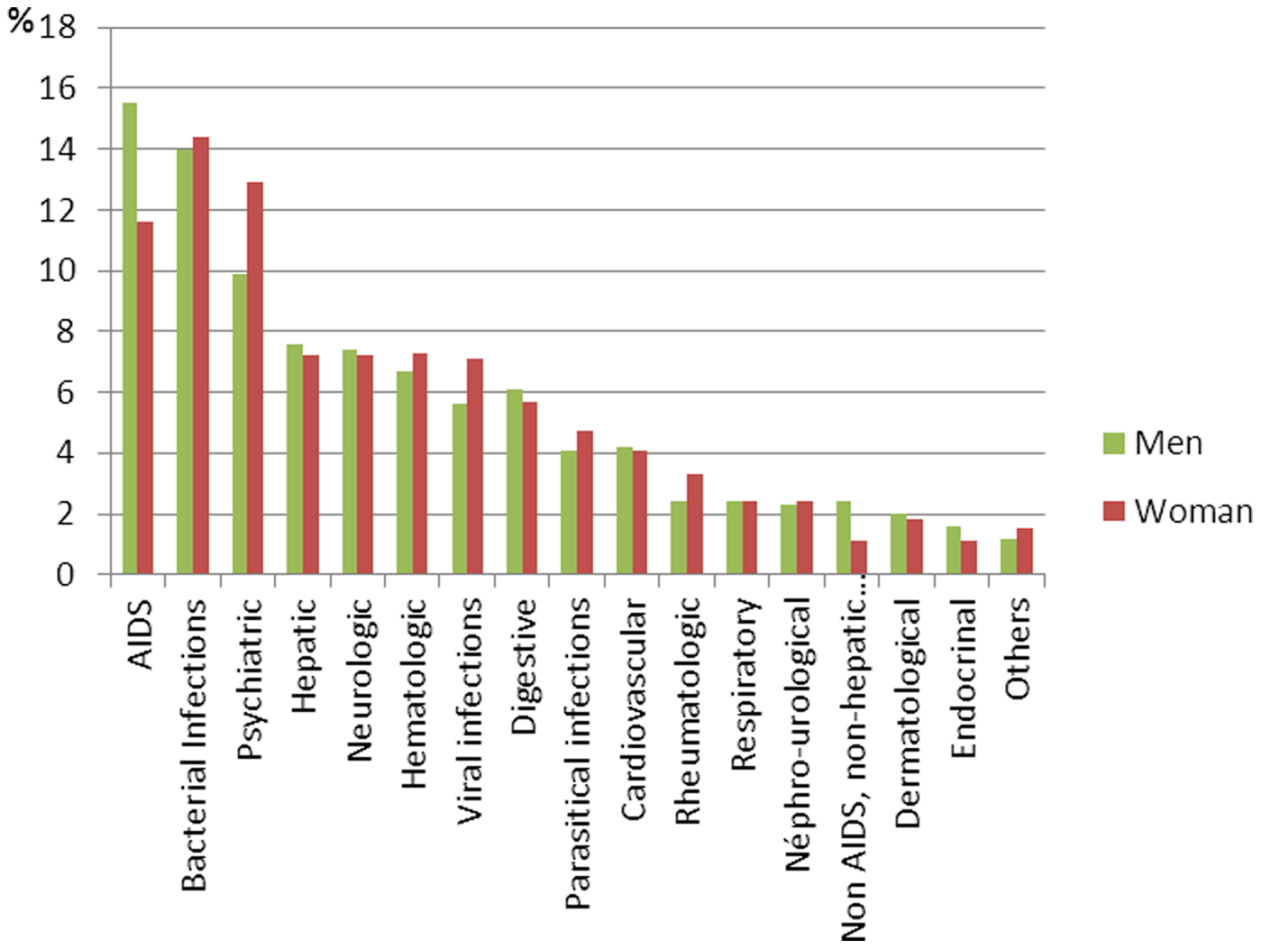
Distribution of severe morbid events according to sex.

Figure 2 represents the different classes of morbid event by sex, displayed in the order of frequency. Aids events remained the most frequent cause of severe morbidity in men (16%, 12% in women), whereas in women they were in second position after bacterial infections (14% in men and women). Except for psychiatric events (13% in women and 10% in men) there were no other differences in the frequency of severe morbid events between men and women.

The most frequent diagnoses in each class of morbid event were the following:

AIDS events (15%) : *Pneumocystis jiroveci* pneumonia, toxoplasma encephalitis, oesophageal candidiasis, cytomegalovirus infections, non-Hodgkin lymphoma, *Mycobacterium avium* infections, Kaposi sarcoma and tuberculosis. There were no cases of invasive cervical cancer. Bacterial infections (14%): pulmonary infections, sepsis, pyelonephritis, syphilis and cutaneous infections. Psychiatric events (11%): depression, addictions (drug, alcohol), psychotic and personality disorders, and suicides. Hepatic events (8%): decompensated cirrhosis and hepatocarcinomas. Neurologic events (7%): epilepsy, neurocognitive disorders and peripheral neuropathy. Haematological events (7%): anaemia, thrombopenia and neutropenia or bone marrow aplasia. Viral infections (6%): respiratory tract infections, herpes zoster infections, herpes simplex infections and diarrheas. Digestive events (6%): diarrhea, pancreatitis, digestive haemorrhage and esogastrtitis or gastroduodenal ulcers. Cardiovascular events (4%): cerebrovascular events, coronary heart disease and myocardial infarction, thromboembolic events, pericarditis and lower limb arteriopathy. Respiratory events (2%): respiratory failure, pleural effusion and asthma. Nephro-urological events (2%): acute renal failure, chronic renal failure interstitial tubulopathy and nephretic colic. Non-AIDS non-hepatic cancers (2%): broncho-pulmonary cancers, Hodgkin's disease, breast cancer, oropharynx cancers and anal cancer. Dermatological events (2%): toxidermitis, seborrheic dermatitis and psoriasis.

**Figure 3:**
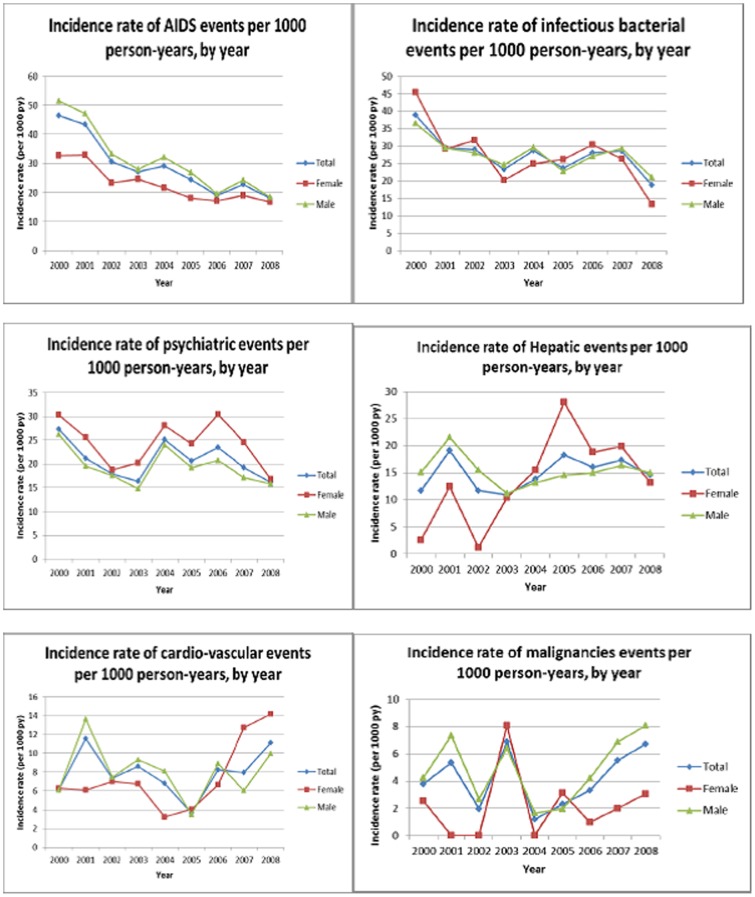
Incidence rates of severe morbid events according to sex (ANRS CO3 Aquitaine Cohort 2000–2008). p_1_ : Poisson regression test for trend, p_2_ : Year*sex interaction test. Fig. 3a. AIDS events, p_1_<0.001, p_2_<0.001. Fig. 3b. Bacterial infections, p_1_<0.01, p_2_ = 0.99. Fig. 3c. Psychiatric events, p_1_ = 0.25, p_2_<0.005. Fig. 3d. Hepatic events, p_1_ = 0.18, p_2_ = 0.48. Fig. 3e. Cardiovascular events, p_1_ = 0.25, p_2_ = 0.83. Fig. 3f. Non-AIDS, non-hepatic malignancies, p_1_ =  0.18, p_2_<0.002.

Yearly incidence rates of the most frequent severe morbid events are represented globally and according to sex (Figure 3). AIDS events significantly decreased over calendar years in men and women, from 47 per 1000 PY in 2000 to 20 per 1000 PY in 2008 (p<0.001). Among non-AIDS defining events, bacterial infections also significantly decreased from 39 in 2000 to 21 per 1000 PY in 2008 (p<0.01), though becoming the first cause of hospitalization in 2008 for men and the third cause for women. Cardiovascular events globally increased from 6 to 13 per 1000 PY. This trend was more important in women (6 to 14/1000 PY) than in men (6 to 10/1000 PY). Other events did not significantly progress through years although the trends within sexes were different: hepatic events were stable in men (15 to 13/1000 PY) and increased in women (2.5 to 11.5/1000 PY), and non-AIDS non-hepatic malignancies increased in men (4 to 7/1000 PY) and remained stable in women (2.5/1000 PY) (Figure 2).

The incidence rate of events decreased through the study period for both groups (with or without cART) but this decrease was more important for the cART group, i.e. from 263.3 per 1000 PY in 2000 to 177.6 in 2008 than the group without cART, i.e. from 261.6 to 201.2 (p<0.001) (results not shown).

### Determinants of severe morbidity

In the multivariate analysis (Table 2), the incidence of severe morbidity was independant of sex (p = 0.23). IDU transmission group, age over 50, HIV RNA over 10,000 copies/ml, CD4 cell count below 500/mm^3^, AIDS stage, hepatitis C co-infection and cardiovascular risk factors such as diabetes, high blood pressure, and tobacco use were all significatively associated with the occurrence of severe morbidity.

**Table 2:** Determinants of severe morbidity according to sex (Generalized estimated equations univariate and multivariate analyses).

Characteristics	Univariate model						Multivariate model	
	Male		Women		Total		Total	
	IRR*	p	IRR	p	IRR	p	IRR [95% CI]	p
**Calendar year**								
2000–2004	1	-	1	-	1	-		
2005–2008	0.82	<0.01	0.98	0.89	0.86	<0.05	0.93 [0.82–1.06]	0.27
**Female sex**					0.96	0.59	1.10 [0.94–1.30]	0.24
**Transmission group**								
Men who have sex with men	0.82	0.07	-	-	1.03	0.72	1.02 [0.84–1.23]	0.85
Heterosexual	1	-	1	-	1	-	1	-
Intravenous drug users	1.56	<0.001	2.43	<0.001	1.91	<0.001	1.40 [1.14–1.72]	<0.01
Others/undetermined	1.21	0.33	1.61	0.11	1.45	<0.05	1.11 [0.80–1.44]	0.46
**Age (years)**								
18–39	1	-	1	-	1	-	1	-
40–49	1.10	0.25	1.35	<0.05	1.17	<0.05	1.02 [0.89–1.17]	0.75
≥50	1.16	0.16	1.42	0.08	1.23	<0.05	1.30 [1.09–1.56]	<0.01
**HIV plasma RNA**								
<500	1	-	1	-	1	-	1	-
500–9999	1.13	0.16	1.25	0.18	1.17	<0.05	1.16 [0.98–1.36]	0.08
10000–99999	1.98	<0.001	2.57	<0.001	2.15	<0.001	1.64 [1.39–1.93]	<0.001
≥100000	5.58	<0.001	6.94	<0.001	5.91	<0.001	2.31 [1.91–2.79]	<0.001
**CD4 (/mm^3^)**								
≥500	1	-	1	-	1	-	1	-
200–499	1.74	<0.001	1.83	<0.001	1.77	<0.001	1.40 [1.20–1.62]	<0.001
50–199	6.10	<0.001	7.83	<0.001	6.52	<0.001	3.59 [2.96–4.35]	<0.001
<50	21.39	<0.001	19.77	<0.001	21.08	<0.001	6.78 [5.32–8.65]	<0.001
**Antiretroviral therapy (cART)**								
No cART	1	-	1	-	1	-	1	-
Protease-inhibitor based cART	1.31	<0.01	1.16	0.38	1.27	<0.01	0.98 [0.83–1.16]	0.84
Other cART	0.78	<0.05	0.82	0.26	0.79	<0.05	0.91 [0.76–1.08]	0.27
**AIDS stage**	5.05	<0.001	5.21	<0.001	5.07	<0.001	2.68 [2.32–3.08]	<0.001
**Dyslipidemia^1^**	1.00	0.96	1.31	<0.05	1.08	0.28	0.97 [0.86–1.11]	0.69
**Diabetes^2^**	1.80	<0.001	2.36	<0.01	1.88	<0.001	1.69 [1.40–2.05]	<0.001
**High blood pressure^3^**	1.32	0.07	1.54	0.12	1.37	<0.05	1.42 [1.12–1.79]	<0.01
**Hepatitis C antibody**	1.61	<0.001	2.44	<0.001	1.80	<0.001	1.43 [1.20–1.70]	<0.001
**Tobcco use (any time)**	1.21	<0.05	1.60	<0.01	1.31	<0.001	1.21 [1.05–1.40]	<0.01

*IRR:I ncidence rate ratio 1.Hyper-cholesterolemia or hyper-triglyceridemia, 2. glycemia >7 mmol/L or any use of antidiabetic drugs, 3. blood pressure ≥14/9 cm Hg or any use of antihypertensive drugs.

Overall, across the study period only 5.7% of patients in 2000 and 6.9% in 2008 was not concerned by any of these detrminants (results not shown).

The variables initially retained in the univariate model and not significantly associated with severe morbidity in the final model were calendar year, ART therapy and dyslipidemia.

### Polypathology

The number of patients with at least one morbid event decreased through years, from 10% in 2000 to 5.2% in 2008 (10.6% to 5.3% in men, 8.4% to 4.9% in women). Among patients with at least one morbid event, the proportion of patients with more than five events increased from 2000 to 2008, from 7.8 to 13.6%. In 2008, 15% of men and 10% of women with at least one morbid event experienced more than five morbid events.

## Discussion

Between 2000 and 2008, a quarter of ANRS CO3 Aquitaine Cohort participants were women. As compared to men, they were younger, more often originating from sub-Saharan Africa, IDU and co-infected by HCV and less likely to receive cART or to present cardiovascular risk factors. The incidence of hepatic and cardiovascular events increased among women over time. In men the incidence of non-AIDS- non-hepatic malignancies and cardiovascular events increased but for the latter this trend was less important than in women.

In accordance with several previously published studies [6], [21]–[24], the number of hospitalized patients decreased in our cohort through the study period. Yearly incidence rates of hospitalization were halved between 2000 and 2008 overall in men and women, decreasing from 147 per 1,000 PA in 2000 to 77 in 2008.

Yearly incidence rates of AIDS events decreased during these years of consolidated cART.

Bacterial infections were the most frequent cause of severe morbidity [21], [25]. Pulmonary infections were indeed a leading diagnosis among bacterial infections, in a cohort in which 24% of patients were HIV-infected through IDU and 55% regular smokers. The overall yearly incidence rates of bacterial infections decreased over time suggesting the long-term effect of immune reconstitution on the incidence of this severe morbidity. However this decrease was more important in women (by 70%) in comparison to men (by 40%). Probably the larger proportion of IDU as a HIV transmission group in women is not a proof of their current drug consumption Tobacco use was more frequent among men than women, overall, 58% of men and 49% of women were current or past smokers. Prevention policies (tobacco cessation counselling, vaccination against influenza and pneumoccal infection), insufficiantly used, should be largely implemented in this population, irrespective of sex and taking into account their specificities [26] in order to reduce their morbidity. It is here noteworthy to point out the potential effect of drug-related harm reduction policies on the decline of the proportion of IDU among men and women across the years between 2000 and 2008.

Hepatic causes of severe morbidity, were globally stable between 2000 and 2008. Women, were more concerned by this morbidity from 2003 onwards, surviving to HIV already seven years after the advent of cART. Indeed, comparatively to the beginning of the study period where HIV-related complications predominated on the other morbid events, in the latest years, women, more often belonging to the IDU transmission group and co-infected with HCV, lived longer to develop hepatic complications such as cirrhosis.

Unlike other morbid events, the overall incidence rates of cardiovascular (CV) morbidities have almost doubled between 2000 and 2008, from 6 to 13 per 1000 PY. This is consistent with the observations of large inter-cohort studies such as D:A:D showing a 26% increase in the risk of myocardial infarction per year of exposure to cART [27] and an increased risk of cerebrovascular events [28]. The higher frequency of risk factors of cardiovascular diseases (dyslipidaemia, diabetes, high blood pressure, tobacco use) in men may be the main reason of this disparity. The yearly incidence rates of cardiovascular events increased more sharply in women especially since 2004, from 3 to 16 per 1000 PY. Recent studies have noted a higher relative risk of myocardial infarction (MI) among HIV-infected women in comparison with men and higher standardized mortality ratio (SMR) in comparison with the general population [29]–[31]. In the French hospital Database on HIV (FHDH), MI SMRs of 1.4 and 2.7 have been reported respectivley for men and women in comparison with the general population [29].

In our study, cardiovascular events are in 2007 and 2008 more frequent in women whereas the frequency of risk factors are higher in men. This is probably due to an unequal treatment of these risk factors between men and women. In the Aquitaine Cohort, the cardiovascular risk factors are equally measured for men and women. Traditionally, men are more often addressed to cardiovascular specialists for screening of CV disease, because the male sex has always been counted like one CV risk factor achieving 50 years of age. So in spite a higher frequency of risk factors (the definition of each disorder includes the treatment of the disorder ex. A man with a lipid lowering drug is counted for having a dyslipidemia, even if his cholesterol or triglyceride plasma rates are in the normal range). This primary prevention policy should certainly explain the lower incidence rate of cardiovascular disease in men comparing to women in the latest years.

Women traditionally not targeted by the cardiovascular prevention policies in part because of the common belief of their natural protection against these diseases have in this HIV-infected population, high burden of risk factors. HIV itself, high rate of smoking (50% vs. 22% in the general French women population [32]), and cART metabolic adverse events (hypercholesterolemia : 33% vs. 15% in the general French women population aged 18–64 years [32]) associated with the ageing of this population and the loss of their hormonal protection with menopause (9% of women were ≥50 years), make women the new victims of these emerging morbidities. The management of cardiovascular risk factors has been shown to have a favorable impact on the incidence of cardiovascular events in recent years [33].This includes the use of lipid-lowering agents, the prescription of PI-free c-ART, and preventive cardiologic monitoring. All these measures are presumeably more often adressed to men than to women so far.

Finally, non-AIDS non-hepatic cancers represented a minor cause of severe morbidity. However their incidence rate increased between 2000 and 2008, from 4 to 7 per 1000 PY. This was particularly true for men (from 4 to 8 per 1000 PY), whereas it was rather stable in women (3 per 1000 PY). Indeed, the risk factors of broncho-pulmonary and oropharynx cancers are more prevalent in men. Moreover, anal intercourse and STIs of anal localization, conventional risk factors of anal cancer are mainly presented by MSMs. This disparity in the incidence rates of cancer between men and women is consistent with the findings of the FHDH reporting a higher incidence of non-AIDS cancers in men, comparing to women [34]. This has also been reported in populations where the higher risk of non-AIDS-defining cancers (NADC) is primarily among males, with HIV-infected women having no higher rates of NADC compared with the overall population [34]–[38].

Unlike the number of patients with severe morbidity and the rate of hospitalization which decreased through our study period, more people living with and in care presented with at least five morbid events in the recent years, reflecting that HIV disease becomes a chronic multisystem condition [39], [40]. A recent american study [41] found polymorbidity more prevalent in women (73%) than in men (67%), mostly due to higher rates of chronic conditions related to higher prevalence of obesity in women. In our study of severe morbidity leading to hospitalization 15% of men and 10% of women experienced more than five morbid events in 2008. These data support the need for early screening of comorbidities in women as well as men, as sex is not a determinant of severe morbidity, unlike the general population where women are less concerned by age-related morbid conditions.

Women were less frequently at the AIDS stage; this might explain why they are less often under cART. The difference between men and women for the cART use tends to decrease across the years. Interestingly there is no difference between men and women in terms of CD4 cells and HIV-RNA.

Globally, IDU transmission group, age over 50 years, HIV RNA over 10,000 copies/ml, CD4 cell count below 500/mm^3^, AIDS stage, hepatitis C co-infection and cardiovascular risk factors such as diabetes, high blood pressure, and tabacco use were significatively associated with severe morbidity, irrespective of sex. Patients with one or more of these characteristics(a vast majority of our cohort) should certainly benefit from a close monitoring, whereas those without these features (less than 10% of our cohort) could be followed once or twice a year, alternately by the general practitioner and a hospital HIV specialist. This is the kind of ground data that has lead to the most recent recommendations for HIV clinical care in France [42]. Furthermore, a CD4 lymphocyte count below 500/mm^3^ remains an important determinant of morbidity as this has been already reported in large cohort collaborations of Western Europe, North America and Australia [43]–[46], as well as in sub-Saharan Africa [47]. This supports the need for early initiation of cART, as recently agreed in France with the recommendation of universal test and treat [42].

As no interaction between sex and these determinants has been found, our data suggest that disparities in severe morbidities such as cardiovascular or hepatic events between men and women are explained by different exposure to risk factors but do not imply different prevention strategies.

Our study had potentiel limitations. The definition of severe morbidity was extented to all clinical diagnoses at hospital discharge. Unlike some other similar studies where the primary cause of hospitalization was used and was categorized based on an organ and disease system coding scheme [48], we have taken into account each different diagnosis present during the hospitalization. This can overestimate the level of severity of these diagnoses as all were not the cause of hospitalization. Their negligence however, would have deprived us from the possibility to thoroughly describe the causes of severe morbid events. Differently to some studies[48], we have excluded hospitalizations of less than 48 hours. This could eliminate a fraction of severe morbid events. This arbitrary decision could counterbalance the previous limitaion by raising the level of severity. This is consistent with the previous study on severe morbidity of the Aquitaine Cohort [21]. Another potentiel limitation of our study is the lack of information on the social class, not collected in the Aquitaine Cohort. Even in a universal care system, social inequalities may explain differences in access to treatment and hospitalization.

Finally, the calendar period of our study was limited to 2008. This paper follows a previous work on the Aquitaine Cohort covering the period 2000–2004 [21]. Validation of severe morbid events is very time-consuming with return to files needed on a vast territory of health care centers in the Aquitaine region (South-Western France). So it was not possible to include completely validated data on the most recent period. We believe however that the main messages would remain true until the most recent years.

In the Aquitaine Cohort, sizeable efforts are allocated for the collection, documentation and uniform classification and coding of morbid events. There is a lack of published studies on the long-term causes of severe morbidity leading to hospitalization in HIV-infected patients, although such information is highly valuable in identifying priorities for case management and improvement of the quality of life of patients with a chronic disease requiring life-long treatment. More collaborative efforts among cohorts should be set up to standardize the collection, documentation and classification of non AIDS-related morbid events, as they are and will remain the largest contribution to the complications of long-term care and life with HIV infection.

Our cohort is to our knowledge the first to report a comprehensive spectrum of women's specificities over a long follow-up period in the consolidated cART era and highlights priorities for improvement in case management not only to prolong survival but also to improve their quality of life.

In conclusion, the morbidity occurring now among HIV infected individuals in long-term care is wide and definitively no longer limited to AIDS-defining events. French women like probably in most industrialized countries, present more often now with complications of viral hepatitis, that should be systematically screened, monitored and managed. Women should also be targeted by prevention policies of cardiovascular events as much as men, by reducing risk factors and enhancing monitoring.

## Supporting Information

Appendix S1 Composition of the Groupe d'Epidémiologie Clinique du SIDA en Aquitaine (GECSA).
(DOCX)
