# Supplementary material for: Severe Morbidity According to Sex in the Era of Combined Antiretroviral Therapy: The ANRS CO3 Aquitaine Cohort
Source: PLoS One. 2014 Jul 30;9(7):e102671. doi: 10.1371/journal.pone.0102671 (PMC4116171; doi:10.1371/journal.pone.0102671)
Supplement: Appendix S1 — Composition of the Groupe d'Epidémiologie Clinique du SIDA en Aquitaine (GECSA). (DOCX) [file pone.0102671.s001.docx]

**Appendix**

Composition of the Groupe d’Epidémiologie Clinique du SIDA en Aquitaine (GECSA)

**Principal investigator:**

Pr F. Dabis

**Scientific committee:**

Prs F. Bonnet, D. Breilh, F. Dabis, M. Dupon, G. Chêne, H. Fleury, D. Malvy, P. Mercié, I. Pellegrin, P. Morlat, D. Neau, JL. Pellegrin R. Thiébaut; Drs S. Bouchet, V. Gaborieau, D. Lacoste, S. Tchamgoué.

**Composition of the GECSA:**

**Epidemiology and biostatistics:** Prs G. Chêne, F. Dabis, R. Thiébaut, Drs M. Bruyand, S. Lawson-Ayayi, L. Wittkop

**Clinical and biological hospital units**

- Bordeaux University Hospital: Pr P. Morlat (Pr F. Bonnet, Drs N. Bernard, M. Hessamfar, D. Lacoste, MA. Vandenhende) ; Pr M. Dupon (Drs FA. Dauchy, H. Dutronc), Pr M. Longy-Boursier (Pr P. Mercié, Drs P. Duffau, J. Roger Schmeltz), Pr D. Malvy (Drs T. Pistone, MC Receveur), Pr D. Neau (Drs C. Cazanave, A. Ochoa, MO. Vareil), Pr JL. Pellegrin (Pr JF. Viallard, Drs C. Greib, E. Lazaro) ; Pr H. Fleury (Pr ME. Lafon, Drs S. Reigadas, P. Trimoulet) ; Pr D. Breilh ; Pr M. Molimard (Drs S. Bouchet, K. Titier) ; Pr JF. Moreau (Dr I. Pellegrin) ; Drs F. Haramburu, G. Miremont-Salamé
- Arcachon Hospital: Dr A. Dupont
- Dax Hospital: Dr Y. Gerard (Drs L. Caunègre, K. André)

- Bayonne Hospital: Dr F. Bonnal (Drs S. Farbos, MC. Gemain)
- Libourne Hospital: Dr J. Ceccaldi (Dr S. Tchamgoué)
- Mont-de-Marsan Hospital: Dr S. De Witte (Dr C. Courtault)
- Pau Hospital: Drs E. Monlun (Dr V. Gaborieau)
- Périgueux Hospital: Dr P. Lataste (Dr JP. Meraud)
- Villeneuve-sur-Lot Hospital: Dr I. Chossat

**Permanent team**

MJ. Blaizeau, M. Bruyand, V. Conte, M. Decoin, J. Delaune, S. Delveaux, F. Diarra, C. D’Ivernois, A. Frosch, S. Geffard, C. Hannapier, S. Lawson-Ayayi, E. Lenaud, O. Leleux, F. Le Marec, J. Leray, I. Louis, G. Palmer, A. Pougetoux, X. Sicard, D. Touchard B. Uwamaliya-Nziyumvira
